# Emerging strategies for monkeypox: antigen and antibody applications in diagnostics, vaccines, and treatments

**DOI:** 10.1186/s40779-025-00660-w

**Published:** 2025-10-22

**Authors:** Wei Wang, Jia-Xiu Li, Si-Qi Long, Zi-Ning Liu, Xi-Peng Li, Zhi-Hang Peng, Ju-Dun Zheng, Yu-Hui Liao

**Affiliations:** 1 Institute for Engineering Medicine, Kunming Medical University, Kunming, 650500 China; 2 Key Laboratory of Infectious Diseases Research in South China, Ministry of Education, Southern Medical University, Guangzhou, 510091 China; 3 National Key Laboratory of Intelligent Tracking and Forecasting for Infectious Diseases, Chinese Center for Disease Control and Prevention, Beijing, 102206 China

**Keywords:** Monkeypox, Monkeypox virus (MPXV), Antigens, Antibodies, Vaccines, Detection, Therapy, Monoclonal antibodies, Mature virion (MV), Enveloped virion (EV)

## Abstract

Monkeypox, a zoonotic illness caused by monkeypox virus (MPXV), has been declared a public health emergency of international concern by the World Health Organization (WHO) on 2 separate occasions. The rapid spread and widespread transmission are closely associated with various proteins involved in the MPXV lifecycle, particularly surface antigen proteins found in mature virion (MV) and enveloped virion (EV), such as A29L, M1R, B6R, and A35R. These antigens are highly conserved in monkeypox virus (MPXV) and vaccinia virus (VACV), possessing cross-protective capabilities that can trigger broad immune protection against multiple orthopoxviruses, including MPXV. Vaccines based on DNA, mRNA, and recombinant proteins, targeting these antigens effectively address the current lack of specific monkeypox vaccines by triggering strong immune responses and ensuring the prevention of monkeypox. Compared to traditional vaccines, multi-epitope vaccines designed using computational tools such as reverse vaccinology and immunoinformatics offer lower development costs and faster validation processes. These multi-epitope vaccines also provide adaptability to mutations in MPXV strains. Additionally, these antigens and corresponding antibodies are useful for diagnosis and therapeutic monitoring, supporting early detection and offering novel treatments for cases resistant to existing antiviral drugs. This review provides a brief summary of recent progress and emerging trends in monkeypox detection, vaccine development, and antibody-based therapy targeting these antigens, offering new insights for monkeypox prevention and control.

## Background

Monkeypox is a zoonotic disease caused by the monkeypox virus (MPXV) [1–4], which includes 2 major genetic lineages: the Congo Basin (Clade I) and West African (Clade II) strains [5–7]. Clade I is associated with more severe disease, higher transmissibility, and significantly higher mortality (> 10%) compared to Clade II (< 1%) [7]. The 2022 outbreak was caused by a subclade of Clade II (lineage B.1). Within just 2 months of the outbreak, 17,300 confirmed and suspected monkeypox cases were reported across 70 countries in all 6 WHO regions [8, 9]. Due to its severity, the World Health Organization (WHO) declared monkeypox a public health emergency of international concern [9, 10]. Although a decline in reported cases followed due to swift global responses, a resurgence led WHO to declare a second emergency on August 14, 2024, prompted by a sharp rise in infections from Clade Ib in the Democratic Republic of the Congo (DRC) and its spread to neighboring countries [11–13]. As of September 14, 2024, the DRC has reported a total of 21,835 suspected MPXV cases, marking a higher case count compared to previous years [4]. Given the significant public health threat, there is an urgent need for advanced and efficient diagnostics, vaccines, and treatments.

Current diagnostics, vaccines, and treatments for monkeypox face several limitations. Established diagnostic methods, such as electron microscopy and polymerase chain reaction (PCR), although accurate, are time-consuming (12 h) and complex in real-world applications. Serological methods are less effective for early diagnosis due to the required antibody development window [14–18]. In the field of vaccines, live replicating vaccinia virus (VACV; ACAM2000) and modified vaccinia Ankara (JYNNEOS) are approved for use by the United States (U.S.) Food and Drug Administration (FDA), are associated with side effects such as fever, allergic reactions, rashes, and myocarditis [19–21]. These live attenuated vaccines include the whole virus, which may trigger immune responses to irrelevant antigens, potentially reducing vaccine specificity and increasing the risk of reinfection or breakthrough cases [22–24]. In addition, there are no drugs specifically designed for monkeypox, and approved treatments rely on repurposed smallpox antivirals [25]. Existing treatments rely on repurposed smallpox antivirals, including Tecovirimat and Brincidofovir [26–29], and also have limitations (such as suboptimal therapeutic efficacy) due to growing MPXV variability and rising drug resistance [30–34]. Thus, while current tools have played a role in combating MPXV outbreaks, there remains a pressing need to develop next-generation diagnostics, vaccines, and treatments.

Antigen and antibody-based approaches have proven essential in combating other infectious diseases, such as coronavirus disease 2019 (COVID-19) and acquired immunodeficiency syndrome (AIDS) [35–38]. These approaches are also highly promising for monkeypox prevention and control. First and foremost, antigen-based detection technologies offer speed and sensitivity, addressing the need for rapid MPXV detection [39]. Vaccines designed using novel antigens or combinations of antigens have demonstrated potential in various animal models [40]. Additionally, monoclonal antibodies (mAbs) and immunoglobulins have shown efficacy against viral infections. The combination of immunoglobulins like vaccinia immunoglobulin (VIG) with antivirals may provide effective options for individuals with drug-resistant MPXV strains or those who are nonresponsive to current treatments [41]. Based on this, antigenic antibodies against MPXV may have new potential for the diagnostics and vaccines of monkeypox, as well as for treatments.

**Fig. 1:**
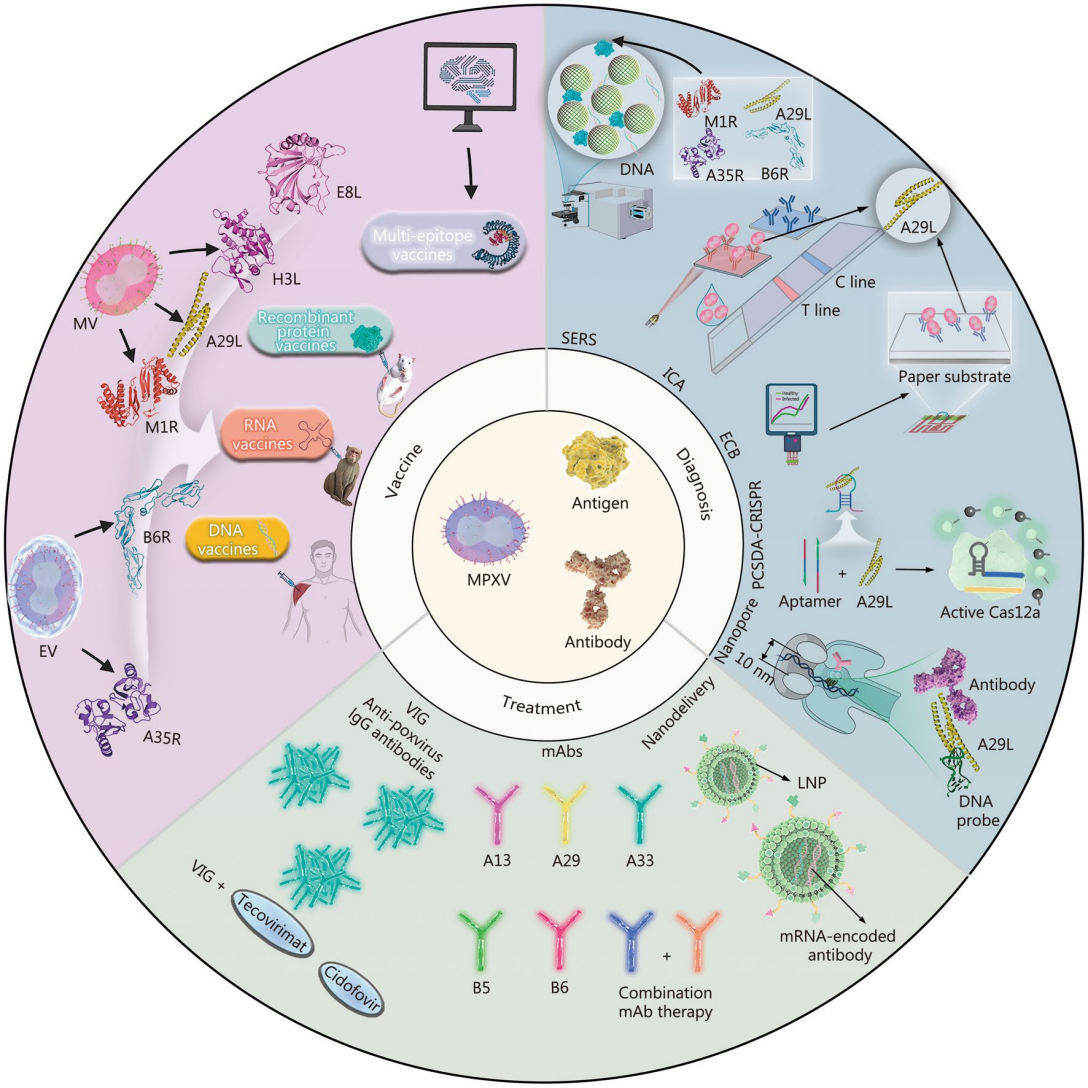
Graphical overview of monkeypox diagnosis, vaccine development, and treatment strategies involving antigens and antibodies. Novel vaccine platforms based on mRNA, DNA, and recombinant protein constructs derived from key antigenic proteins on the surface of mature virion (MV) and enveloped virion (EV), such as A29L, M1R, and A35R, have shown strong immunogenicity and protective efficacy in both murine and non-human primate animal models. Computational tools in immunoinformatics enable the selection of highly antigenic cytotoxic T lymphocyte (CTL), helper T lymphocyte (HTL), and B cell epitopes for designing multi-epitope vaccines, resulting in lower development costs and faster validation processes. Advances in diagnostic technologies focus on antigen recognition strategies, including surface-enhanced Raman spectroscopy (SERS), immunochromatographic assays (ICA), electrochemical biosensors (ECB), nanopore sequencing, and a combined technique involving positional linker-induced cascade chain displacement amplification with CRISPR/Cas12a (PCSDA-CRISPR). Regarding treatment strategies, a synergistic approach combining vaccinia immunoglobulin (VIG) with specific antiviral agents such as Tecovirimat and Cidofovir has been proposed. Monoclonal antibodies (mAbs) targeting antigens such as A29 and B6 demonstrated clinical potential, and lipid nanoparticle (LNP)-based delivery systems have been introduced to improve antibody delivery efficiency

Recently, extensive research has explored the mechanisms of MPXV antigens and antibodies and their application in diagnostics, vaccines, and treatments. Despite these advancements, a comprehensive review remains lacking. Nevertheless, a thorough overview of this field is still lacking. This article provides an overview of the mechanisms of action of MPXV antigens and antibodies and summarizes progress in developing antigen- and antibody-based strategies for monkeypox diagnostics, vaccines, and treatments (Fig. 1). It also outlines promising directions for future research to support improved monkeypox control efforts.

## Monkeypox infection and host immunity

### Monkeypox infection

Understanding the life cycle of MPXV is essential for the development of diagnostics, vaccines, and treatments. The process of MPXV infection can be divided into three stages: 1) viral invasion, 2) viral replication and synthesis, and 3) viral assembly, maturation, and release [42].

Two types of infectious particles are produced in the early stages of MPXV infection: the mature virion (MV) and enveloped virion (EV) (Table 1) [14, 43–56]. These particles differ in envelope composition, surface proteins, and entry mechanisms. MV, with its single-membrane structure, can enter cells through membrane fusion or low-pH endocytosis [57]. In this process, MPXV antigens such as A29, H3, E8, and M1 appear to be important [43–45]. Glycosaminoglycans (GAGs), including heparin, acetylheparin sulfate, and chondroitin sulfate, also facilitate viral attachment to host cells [58]. The A29, H3, and E8 proteins may promote viral adhesion by binding to heparan sulfate or chondroitin sulfate [43–45]. It is worth mentioning that Shi et al. [43] proposed a model for MPXV entry into host cells based on the interaction between A29 and GAGs: 1) viral particles attach to the host cell surface when the A29 protein binds to acetylheparin sulfate; 2) host cell surface protease initiates fusion of the viral and host cell membranes; 3) viral particles enter the host cell. In addition to GAG-mediated binding, MPXV may also attach to non-GAG molecules on the cell surface [59, 60]. After viral attachment, the entry/fusion complex (EFC) plays a significant role in endocytosis or membrane fusion. The L1 protein, a surface antigen on VACV, is a key component of the viral entry machinery and may cooperate with EFC and F9 proteins to mediate viral entry [46]. Viral particles lacking L1 can attach to the cell surface but fail to deliver their cores into the cytoplasm, which blocks subsequent replication [46]. Since orthopoxviruses are highly conserved, MPXV antigenic protein M1 may function similarly during viral entry [61].

**Table 1:** MPXV main antigen proteins

Protein localization	Protein	Role in the replication cycle	Protein function	Application potential	References
MV	A29	Attachment	Binding to heparan sulfate promotes viral attachment to the cell surface	Diagnostic biomarker; Therapeutic monoclonal antibody target	[14, 43, 52, 54]
	H3	Attachment	Binding to heparan sulfate facilitates viral cell attachment	Vaccine target; Therapeutic monoclonal antibody target	[44, 49, 52]
	E8	Attachment	Chondroitin sulfate binding mediates viral attachment	Vaccine target; Therapeutic monoclonal antibody target	[45, 50, 52]
	M1	Entry	Potential interaction with EFC may facilitate viral entry into host cells	Diagnostic biomarker; Vaccine target; Therapeutic monoclonal antibody target	[14, 46, 51, 52]
	A13	Virion maturation	Promotes viral maturation	Therapeutic monoclonal antibody target	[47, 53]
EV	B6	Assembly/spread	May facilitate the rupture of the EV membrane, enabling MV entry into cells; Potential interaction with A34/A35 may promote proper IEVs assembly	Diagnostic biomarker; Vaccine target; Therapeutic monoclonal antibody target	[14, 48, 52, 56]
	A35	Assembly/spread	May interact with A34 and B6 to facilitate proper assembly of intracellular enveloped virions	Diagnostic biomarker; Vaccine target; Therapeutic monoclonal antibody target	[14, 48, 52, 55]

*MPXV* monkeypox virus, *MV* mature virion, *EV* enveloped virion, *EFC* entry/fusion complex, *IEVs* intracellular enveloped viruses

In contrast, EV has a double-membrane structure. The outer membrane must rupture before or during infection for the internal MV to fuse with the plasma membrane or endocytic vesicles [62]. The antigenic protein B5 on EV may play a critical role in this process by promoting the outer membrane through interactions with GAGs [63, 64]. Due to the high homology between the B5 protein and the MPXV B6 protein, B6 may also assist viral entry through a similar mechanism [61]. The rupture of the EV membrane further enables MV entry by destabilizing the acidic environment of endocytic vesicles [62]. Overall, MV antigens are central to the invasion process, although the precise mechanisms of several antigens remain unclear and require further study.

Once MV or EV enters the host cell, the viral core (composed of the central viral genome, nuclear envelope, and nucleocapsid) is transported along microtubules to the nuclear periphery, and initiates the process of decapsidation. Nucleocapsid proteins are ubiquitinated and degraded by proteasomal enzymes as part of the decapitation pathway [65, 66]. Following uncoating, the MPXV genome undergoes efficient replication within specialized replication factories. Numerous endoplasmic reticulum (ER) membranes surround the replication factory, wherein the ER plays an essential role in synthesizing viral membrane proteins. These proteins, along with other viral structural components, are transported to the replication sites to encapsulate the core genes and form crescent-shaped structures [67–69].

Within the replication factory, the MPXV antigenic protein A13L is critical for the transition of immature virions (IVs) into intracellular mature virions (IMVs). This transformation requires proteolytic cleavage and condensation of viral core proteins. Inhibition of A13L protein expression reduces the production of infectious virus by up to 1000-fold, reflecting its importance in viral maturation [47]. In addition, the antigenic proteins A33, A34, and B5 can co-localize with Golgin-97 in the ER near the nucleus [48]. Through transient glycoprotein interactions, these proteins are transported to the trans-Golgi network (TGN), which supports proper assembly of intracellular enveloped viruses (IEVs) [48]. Following IMV formation, a fraction of these particles exit the replication factory and become wrapped by the TGN as IEVs. Proteins A33, A34, and B5 are efficiently delivered to the TGN during this process, promoting IEV synthesis and release [48, 70, 71]. Because these proteins are highly conserved among orthopoxviruses [61], similar interactions likely occur in MPXV infection and may be essential for viral maturation and release. After IEVs reach the cell periphery, they fuse with the plasma membrane and release EVs with a double-membrane structure. Thus, antigens on EV are important not only for viral entry but also for maturation and release. However, further experimental studies are needed to fully clarify the mechanisms of action of these antigens.

### Host immunity

When MPXV enters the body, it triggers the host’s immune system, which generates a range of responses to combat the virus. Immune cells and effector molecules, including T cells, B cells, and antibodies, play crucial roles in this defense. Simultaneously, MPXV promotes its replication in host cells through various immune evasion strategies.

Following entry into host cells, MPXV-derived pathogen-associated molecular patterns are recognized by intracellular Toll-like receptors (TLRs), which initiate innate immune responses [72]. Concurrently, antigenic proteins from VACV undergo proteasomal degradation within antigen-presenting cells (APCs) or are internalized via endocytosis for lysosomal degradation. Processed peptides subsequently bind to major histocompatibility complex (MHC) class I or II molecules and are presented on the cell surface, thereby eliciting adaptive immune responses [73–75]. Given the high structural and functional similarity between MPXV and VACV, MPXV antigens likely follow similar pathways to activate adaptive immunity.

Immune cells are essential components of host defense against viral infections. Innate immune cells form the first line of defense and are also primary targets of viral attack. In the early stages of MPXV infection, monocytes are rapidly recruited to the infection sites and become early targets of viral entry [76]. MPXV and other orthopoxviruses can also infect macrophages and use them to facilitate viral dissemination [76]. Natural killer (NK) cells are another important component of innate immunity. These cells express chemokine receptors that guide them to the infection sites. Although NK cell numbers increase significantly in MPXV-infected individuals, their function is impaired [77]. Specifically, NK cells show reduced chemotaxis and degranulation, decreased expression of chemokine receptors such as C-X-C chemokine receptor 3 (CXCR3), C-C chemokine receptor 5 (CCR5), CCR6, and CCR7, and a diminished ability to secrete tumor necrosis factor-α and interferon (IFN)-α [77], impairing NK cell function. Lymphocytes, including T and B cells, are central to adaptive immunity. CD4^+^ T cells enhance the differentiation and recall of B cells into antibody-secreting cells [78], and their abundance is critical for generating protective antibody responses against severe MPXV infection. In simian immunodeficiency virus (SIV)-infected rhesus macaques, CD4^+^ T cell count < 300 cells/mm^3^ is insufficient for effective protection following vaccination [79]. In addition to supporting antibody production, T cells exert direct antiviral effects. Activated CD8^+^ T cells can eliminate virus-infected monocytes through the release of perforin and granzyme, thereby reducing viral spread, as demonstrated in a VACV mouse model [80]. B cells contribute by producing antibodies that agglutinate, opsonize, and neutralize viral particles, immobilize complement, and mediate antibody-dependent cytotoxicity (ADCC) [81]. The importance of T and B cell responses, along with antibody-mediated immunity, is well established. Since CD4^+^ and CD8^+^ T cell epitopes are highly conserved across poxviruses [82], similar immune mechanisms may be involved in MPXV infections. However, the exact pathways require further study. Although T cells play a crucial role in host immunity, MPXV197, a member of the MPXV B22 protein family, may facilitate immune evasion by interfering with the T cell receptor (TCR) and impairing MHC-dependent T cell activation [83]. Other types of immune cells, including dendritic cells, are also altered during MPXV infection [84, 85]. A comprehensive investigation of the roles of different immune types is necessary to better understand immune responses and develop targeted immunotherapies.

Immune effector molecules also influence disease severity and progression in MPXV infection. The presence of MPXV in cells leads to elevated levels of specific neutralizing antibodies in vivo. IgM antibodies play an important role in primary immune responses, while IgG antibodies play a dominant role in secondary immunity by reflecting the activity of IgG^+^ memory B cells [76]. A 2003 study of MPXV-infected patients found that individuals with moderate or severe disease exhibited higher anti-orthopoxvirus IgM titers than those with mild disease. This suggests that IgM responses may serve as a marker of disease severity [86]. Some antibodies rely on complement for their activity, such as anti-A35R antibodies [87]. The complement system, a critical component of innate immunity, is activated early during MPXV infection. Proteomic analysis of bronchoalveolar lavage fluid from MPXV-infected rhesus monkeys revealed that complement-related genes were among the most significantly altered inflammatory genes [88]. Certain MPXV strains, particularly those in evolutionary Clade I, express the monkeypox complement inhibitor MOPICE, which binds to human C3b/C4b, blocking complement activation and supporting viral immune escape [89]. Further elucidation of the roles of immune effector molecules in MPXV infection will contribute to the development of more effective therapeutic and preventive strategies and offer new insights into the design of interventions against monkeypox (Fig. 1).

## Novel diagnostic techniques based on monkeypox antigen

Although nucleic acid-based tests remain the “gold standard” for monkeypox detection, they often require nucleic acid enrichment, which is time-consuming and technically complex. In contrast, antigen-based assays offer a faster and potentially valuable alternative for the rapid MPXV diagnosis [90], which is critical for early detection and control (Fig. 1).

### Surface-enhanced Raman spectroscopy (SERS)

SERS has received growing attention for its advantages, including simple operation, rapid processing, high sensitivity, high specificity, and low cost [91]. Several MPXV antigenic proteins play key roles in host-virus interactions. For example, A29L binds to cell surface heparan sulfate to facilitate viral attachment [43], while M1R and B6R are involved in host cell entry [46, 61, 63]. Additionally, B6R and A35R are closely associated with viral maturation [48]. These 4 proteins are important targets of neutralizing antibodies and are useful for early diagnosis of MPXV. Zhang et al. [14] developed a rapid SERS-based detection technique for both the MPXV gene and 4 antigenic proteins (A29L, A35R, M1R, and B6R). This method addressed the difficulty of capturing signals due to the mismatch between virus size and SERS “hot spots” by employing silver nanoparticles co-precipitated with calcium ions in the presence of iodide ions as the substrate. Combined with principal component analysis, the technique was able to differentiate protein-specific SERS profiles in serum within 5 min. In conclusion, the SERS-based assay for MPXV antigenic proteins demonstrates strong potential for rapid monkeypox diagnostics due to its low detection threshold and short assay time. Although silver nanoparticles are beneficial in SERS biosensors due to their high extinction coefficients and strong scattering properties, their use is limited by toxicity caused by oxidative reactions [92]. Bimetallic nanomaterials, particularly gold-silver (Au/Ag) alloys, offer a promising alternative because of their chemical and thermal stability, low density, and reduced toxicity [93]. These alloys warrant further exploration for the development of improved SERS biosensors.

### Immunochromatographic assays (ICA)

ICA is a widely used immunological technique with significant potential for MPXV detection. Their main advantages include rapidity, ease of use, simple design, and low cost [94]. Commercially available Orthopox BioThreat Alert test kits that utilize VACV antibodies have demonstrated the ability to reproducibly detect VACV or MPXV at 10^7^ PFU/ml, with results readable within 15 min [95]. Ye et al. [96] developed a colloidal gold nanoparticle (AuNP)-based ICA for MPXV detection. The assay used the A29 17–49 peptide to generate monkeypox-specific antibodies through mouse hybridoma technology. This method showed high specificity and sensitivity. However, colloidal gold-based ICA still faces limitations such as insufficient sensitivity and limited capacity for quantitative detection. To address these challenges, researchers are developing strategies to integrate higher-sensitivity optical signals into the ICA system [97]. Fluorescent lanthanide complexes are promising for this purpose due to their long fluorescence lifetime, large Stokes shift, and narrow emission peaks, which help reduce background interference and improve assay speed, sensitivity, and specificity. Yan et al. [98] developed a time-resolved fluorescence immunoassay (TRFIA) for the MPXV A29 protein using europium-based luminescent microspheres. The system produced results within 15 min, with a detection limit of 20 pg/ml and high accuracy in real sample testing. In this system, test strips are used in a double antibody sandwich format. Antigen immunogenicity was enhanced by coupling A29 Aquifex aeolicus lumazine synthase (AaLS), forming a nanoparticle antigen (AaLS-A29). This approach led to the generation of 2 high-affinity antibodies (MXV 14 and MXV 15) targeting A29, further improving detection performance. Quantum dots (QDs) are another class of luminescent materials that have attracted attention due to their strong fluorescence intensity, high stability, and narrow emission spectra. Sun et al. [94] designed an ultrasensitive fluorescence ICA based on multilayered QD nanobeads to detect MPXV A29L in throat swabs. The assay required 20 min for optimal performance and achieved a detection limit of 5 pg/ml. Like the TRFIA approach, this method used a double antibody sandwich design. It also incorporated mRNA immunization and high-throughput single B-cell sequencing to rapidly identify specific antibodies against the MPXV A29L protein (M53 and M78), demonstrating high diagnostic potential. In conclusion, ICA systems with single-signal outputs provide rapid, sensitive, and stable detection of MPXV antigens. The integration of advanced optical signals can further reduce detection thresholds and enhance sensitivity. Emerging fluorescent material, such as aggregation-induced emission luminogens, has already been applied in lateral immunoassay platforms. Their high brightness, large Stokes shift, high sensitivity, and photostability make them promising candidates for future monkeypox antigen detection systems [99–101].

Compared to single-signal systems, which often require more complex instruments for signal interpretation, dual-signal output systems offer greater flexibility and practicality for field testing. These systems meet the needs for rapid screening of MPXV in resource-limited settings and highly sensitive quantitative detection in primary healthcare facilities [102]. Yang et al. [102] developed a lateral flow immunoassay (LFIA) using multilayer SiO_2_-Au core double QD shell nanocomposites, which provide strong colorimetric and enhanced fluorescent dual-signal outputs. This method enables both qualitative sensing and quantitative analysis of MPXV within 15 min. It also demonstrated low detection limits (0.500 and 0.021 ng/ml, respectively) and high sensitivity in both colorimetric and fluorescent modes. While the sensitivity in colorimetric mode was comparable to that of commercially available AuNP-based LFIA methods, the fluorescence mode offered a 238-fold increase in sensitivity, highlighting the strong potential of this approach for MPXV detection. SERS technology also contributes significantly to dual-signal systems by providing fingerprint signals with high photobleaching stability, making it suitable for ultra-sensitive quantitative detection on ICA test strips. Yu et al. [97] developed nanosheets with combined colorimetric and SERS-enhancing activity for MPXV detection using MoS_2_@Au-Au tags. This method enabled rapid, sensitive, and convenient detection of the MPXV A29 antigen across a variety of conditions, offering both visual readouts and SERS analysis. The method achieved limits of detection (LOD) of 0.2 ng/ml in colorimetric mode and 0.002 ng/ml in SERS mode, exceeding the sensitivity of conventional AuNP-based ICA by approximately 5-fold and 500-fold, respectively. Furthermore, it demonstrated excellent performance in detecting MPXV in spiked pharyngeal swab samples, supporting its strong potential for clinical application. In conclusion, the dual-signal output ICA systems hold significant promise for rapid MPXV antigen diagnosis. Their ability to switch between modes according to testing requirements, combined with broader applicability, high sensitivity, specificity, and reproducibility, makes them highly suitable for diverse diagnostic scenarios.

### Electrochemical biosensing technology

Electrochemical biosensors offer advantages such as short testing times, portable instruments, and miniaturized equipment. These features can reduce the required sample volume and eliminate or minimize sample pretreatment, making them suitable for rapid on-site detection [103]. This has significant implications for developing diagnostic technologies targeting MPXV antigens. Chandran et al. [103] developed an electrochemical method for MPXV A29 antigen detection by electro-depositing a molybdenum dioxide (MoO_3_)-graphene quantum rods composite onto a flexible paper-based electrode using a one-pot electro-deposition technique. Under optimal conditions, this electrochemical immunosensor achieved a LOD of 0.534 nmol/l and a sensitivity of 4.512 μA. In human serum samples, it maintained a low LOD of 0.685 nmol/l and a sensitivity of 4.49 μA. The strategy is simple, easy to implement, and highly efficient, offering significant advantages for early MPXV diagnosis. In addition to these materials, laser-scribed graphene (LGS) has attracted interest in sensor development due to its large surface area, high conductivity, low cost, and simple fabrication operation. For instance, de Lima et al. [104] created a portable electrochemical immunosensor targeting the A29 protein using porous LGS electrodes. This device is easy to fabricate and operate, does not require specialized personnel or complex instrumentation, and can be powered by a miniature potentiostat connected to a smartphone.

### Aptamer-based optical biosensors

Compared to traditional protein-based recognition elements, such as antibodies, nucleic acid aptamers can be structurally engineered to improve binding affinity and specificity, enabling precise detection of pathogen proteins [105]. Han et al. [106] developed a CRISPR/Cas12a-mediated aptasensor for detecting the monkeypox A29 antigen. This system employed 2 high-affinity aptamers, A29-Apt41 and A29-Apt12, capable of recognizing the A29 protein at picomolar levels. A proximity ligation assay was used to form a sandwich coupling switch (SCS) complex between the 2 aptamers and the target protein. This complex converted the recognition event into a measurable fluorescent signal and achieved a LOD of 0.284 ng/ml. This method presents a promising approach for the early diagnosis of MPXV based on antigen recognition.

### Nanopore detection

Nanopore technology has gained attention for its powerful capabilities in molecular sensing, chemical analysis, and single-molecule detection, making it a valuable platform for monkeypox antigen assays [107]. The MPXV A29 antigen, a highly conserved surface envelope protein, poses a challenge for selective detection due to its strong homology with the A27 protein of VACV. Cai et al. [107] developed a nanopore sensor using a customized DNA molecular probe that targets the A29L protein. This system significantly improved single-molecule resolution and enabled selective detection of A29 directly in human body fluids with a LOD of 11 fmol/l. However, the requirement for a long incubation period of 2 h limits its suitability for rapid on-site diagnostics. Further clinical validation and optimization are needed to improve its field applicability.

In summary, MPXV antigens have been successfully applied in a wide range of detection technologies. Antigen-based detection enables fast and cost-effective diagnosis, which is crucial for early MPXV identification, especially in low-resource settings (Table 2) [14, 94–98, 102–104, 106, 107]. These methods show promising reliability for rapid testing. Although antigen-based diagnostics generally exhibit lower sensitivity than PCR, their shorter turnaround time renders them valuable for point-of-care testing. According to the WHO Target Product Profile for orthopoxvirus antigen-detecting assays, the minimum acceptable LOD is 10⁶ PFU/ml, while the preferred limit is 10^4^ PFU/ml or lower, with an optimal test duration of 20 min or less [108]. Encouragingly, many current technologies meet these benchmarks, demonstrating LOD of 10^4^ PFU/ml or better within 20 min. However, additional validation in complex clinical samples is needed to confirm their effectiveness under real-world conditions. The emergence of dual-mode antigen detection assays highlights operational flexibility, allowing users to select the appropriate detection mode based on specific needs [97, 102]. This feature enhances their potential value in a resource-limited environment. Despite these advances, antigen-based diagnostics for monkeypox face several limitations. First, their sensitivity must be improved to increase diagnostic accuracy. Second, the number of validated MPXV antigens remains limited, and expanding the antigen repertoire is essential to further improve assay performance. Third, most current assays lack large-scale clinical trials to confirm their diagnostic utility. Notably, most of the described techniques rely on protein-based recognition elements, primarily utilizing the specific binding between antigens and antibodies. A smaller number apply aptamer-based recognition strategies, which represent an innovative direction for MPXV diagnostics and deserve future exploration [109]. In addition, nanomaterials such as metal nanoparticles and carbon-based compounds have already been employed in diagnostic tools for infectious diseases [93], including monkeypox. These materials offer broad potential and should be further investigated in future studies.

**Table 2:** Novel monkeypox antigen-based diagnostic technologies

Detection platform	Name	Limit of detection	Assay time (min)	Laboratory dependency	References
Surface-enhanced Raman scattering	Surface-enhanced Raman spectroscopy (SERS)	5 ng/ml (MPXV protein); 100 copies/ml (MPXV nucleic acid)	2	Specialized laboratory equipment	[14]
Immunochromatographic assays (ICA)	Tetracore Orthopox BioThreat® antigen detection assay	10⁷ PFU/ml (VACV and MPXV)	15	Basic laboratory equipment	[95]
	Gold-based colloidal gold ICA	50 pg/ml (A29 protein)	10–15	Basic laboratory equipment	[96]
	TRFIA-based point-of-care diagnostic system for MPXV detection	20 pg/ml (A29 protein)	≤ 15	Basic laboratory equipment	[98]
	Ultrasensitive fluorescent ICA-based on multilayer quantum dot nanobeads	5 pg/ml (A29 protein); 1 × 10^4^ PFU/ml (inactivated MPXV)	≤ 20	Basic laboratory equipment	[94]
	Si-Au/DQD-based lateral flow immunoassay	0.5 ng/ml [A29 protein (colorimetric mode)]; 0.0021 ng/ml [A29 protein (fluorescence mode)]	15	Basic laboratory equipment	[102]
	Colorimetric/surface-enhanced Raman scattering dual-signal co-enhanced ICA	0.2 ng/ml [A29 protein (colorimetric mode)]; 0.002 ng/ml [A29 protein (fluorescence mode)]	≤ 20	Basic laboratory equipment	[97]
Electrochemical biosensing technology	Label-free electrochemical immunoprobe	0.524 nmol/L (in PBS buffer); 0.685 nmol/L (in human serum samples)	30	Specialized laboratory equipment	[103]
	Electrochemical paper-based nanobiosensor for rapid and sensitive detection of MPXV	3.0 × 10^-16^ g/ml (A29 protein); 7.8 × 10^-3^ PFU/ml (MPXV)	15	Basic laboratory equipment	[104]
Aptamer-based optical biosensor	PCSDA-CRISPR assay	0.284 ng/ml (A29 protein)	N/A	Specialized laboratory equipment	[106]
Nanopore sensing	Selective single-molecule nanopore detection of monkeypox A29 protein directly in biofluids	11 fmol/L (A29 protein)	N/A	Specialized laboratory equipment	[107]

*MPXV* monkeypox virus, *VACV* vaccinia virus, *TRFIA* time-resolved fluorescence immunoassay, *Si-Au/DQD* Silica-Au core dual-quantum dot shell nanocomposite, *N/A* not applicable, *PCSDA-CRISPR* proximity ligation assay-induced cascade strand displacement and amplification reactions integrated with CRISPR/Cas12a assay, *PBS* phosphate-buffered saline

## Monkeypox antigen-based vaccines

Vaccines are widely recognized as the most effective tools for preventing and potentially eradicating infectious diseases [110–113], and they play a critical role in the fight against MPXV. Although 2 live attenuated vaccines (JYNNEOS and ACAM2000) have been officially approved for monkeypox prevention, their limitations make them insufficient for addressing current outbreak conditions [25, 114]. In contrast, antigen-based vaccines, including DNA, mRNA, recombinant proteins, and multi-epitope formats, are capable of training the immune system to recognize foreign antigens and trigger both humoral and cellular responses [79, 115]. This section reviews recent advances and emerging trends in monkeypox antigen-based vaccine development.

Selecting appropriate protective antigens is a key step in vaccine design. Studies have shown that approximately 20–30 of the 190 proteins encoded by the MPXV genome are major targets for vaccine research [116, 117]. These targets mainly include surface antigenic proteins found on the infectious forms of the virus: MV and EV. The MV-associated proteins include A29L, M1R, E8L, and H3L, which correspond to A27L, L1R, D8L, and H3L in VACV. EV-associated proteins include A35R and B6R, which correspond to A33R and B5R in VACV. These proteins are involved in viral attachment, host cell entry, and transmission [57, 118–123]. Animal studies have shown that these antigens elicit strong neutralizing antibodies and antiviral T cell responses in both mice and crab-eating monkeys, protecting MPXV and VACV [44, 124–127]. Their high sequence conservation across MPXV, VACV, and Variola virus (VARV) makes them ideal candidates for a cross-protective vaccine designed to confer broad immunity against multiple orthopoxviruses, including MPXV [116, 128]. Additionally, using a combination of MV and EV antigens has been shown to elicit broader immune responses than using either alone [127, 129–131]. As a result, many next-generation monkeypox vaccines adopt a multivalent design strategy that includes both types of antigens.

### DNA vaccines

DNA vaccines offer significant advantages for monkeypox prevention due to their rapid design, ease of production, and excellent thermal stability (Table 3) [131–133]. These vaccines introduce eukaryotic expression vectors containing selected antigens into host cells. Once inside the host, these vectors are transcribed and translated into antigenic proteins, which then stimulate the immune system. The host recognizes these proteins as foreign and mounts a response involving cytotoxic T cell activation and antibody production [116].

**Table 3:** DNA and mRNA vaccines

Type	Name	Antigen		Model	Phase	References
		MV	EV			
DNA vaccine	4pox DNA vaccine	A27L, L1R	A33R, B5R	Macaques, mice	N/A	[131, 132]
	N/A	A27, L1, F9, H3	A33, B5, A56, A4	Macaques	N/A	[133]
RNA vaccine	mRNA-A-LNP, mRNA-B-LNP	A29L, M1R	A35R, B6R	Mice	N/A	[23]
	N/A	A29L, M1R	A35R, B6R	Mice	N/A	[136]
	mRNA-1769	A29L, M1R	A35R, B6R	Macaques	Phase I/II	[137]
	BNT166a	H3, M1	A35, B6	Mice, macaques	Phase I/II	[40]
	BNT166c	M1	A35, B6			
	AR-MPXV5	M1R, E8L, A29L	A35R, B6R	Mice	N/A	[138]
	AR-MPXV4a	M1R, E8L, A29L	B6R			
	AR-MPXV4b	E8L, A29L	A35R, B6R			
	AR-MPXV3	E8L, A29L	B6R			
	MPXV-E2	N/A	A35R, B6R	Mice	N/A	[139]
	MPXV-M2	H3L, M1R	N/A			
	MPXV-M4	A29L, E8L, H3L, M1R	N/A			
	MPXV-EM6	A29L, E8L, H3L, M1R	A35R, B6R			
	Rmix4	M1, A29	A35, B6	Mice	N/A	[52]
	Rmix6	M1, H3, A29, E8	A35, B6			
	VGPox	M1R	A35R	Mice	N/A	[140]
	MPXVac-097	A29L, E8L, M1R	A35R, B6R	Mice	N/A	[141]
	LBA	A29L	B6R	Mice	N/A	[142]
	LAM	M1R	A35R			
	LBAAM	A29L, M1R	B6R, A35R			
	MPXfus, MPXmix	A29L, M1R	A35R, B6R	Mice	N/A	[143]
	ALAB-LNP, 4Sin-LNP	A27, L1	A33, B5	Mice or rats	N/A	[144]

*MV* mature virion, *EV* enveloped virion, *N/A* not applicable, *LNP* lipid nanoparticle, *MPXV* monkeypox virus

Surface proteins from MV and EV are ideal for DNA vaccine design. A27L and L1R (from MV) facilitate viral attachment and entry, while A33R and B5R (from EV) contribute to viral spread. Hooper et al. [132] developed a quadrivalent DNA vaccine (4pox) containing A27L, L1R, A33R, and B5R. In macaques immunized via gene gun, the vaccine induced neutralizing antibodies against all 4 antigens and conferred protection against challenge with the MPXV Zaire-79 strain. However, the animals continued to shed the virus [131]. In mice challenged with 2 × 10^6^ PFU of the VACV strain IHD-J, all recovered well after vaccination with this vaccine using a skin electroporation device [132].

Additional MV proteins, such as F9 and H3, are also important targets. F9 facilitates host cell entry [134], and H3 binds to heparan sulfate on the cell surfaces, plays a significant role in the formation of mature viral particles and viral infectivity both in vitro and in vivo [44, 135]. Key EV proteins include A56 and A4. A56 is associated with viral entry and membrane fusion, while A4 enhances cytotoxic T lymphocyte (CTL) responses [119, 133]. Hirao et al. [133] tested a multivalent DNA vaccine comprising 8 antigens: A27, F9, H3, L1, A33, B5, A56, and A4. Macaques vaccinated with this formulation were administered a lethal dose of MPXV Zaire-79. All vaccinated animals produced neutralizing antibodies and survived, while most control animals immunized with an empty vector experienced significant weight loss. Only one control animal survived and still showed disease symptoms. The study also compared the intradermal and intramuscular administration routes. Results showed that skin-based immunization generated stronger antibody responses to all antigens and offered stronger immune protection, potentially due to the induction of a T helper 2 cell immune response [133].

In summary, DNA vaccines based on VACV antigens demonstrate excellent stability and tolerability, and they are capable of preventing severe MPXV infections. However, these vaccines face certain limitations. Firstly, their immunogenicity is generally weaker than that of traditional vaccines, particularly in terms of antibody production. To address this, suitable delivery methods such as intradermal injection combined with electroporation may be necessary to improve immune responses [116, 133]. Secondly, there is a potential risk of integration into the host genome. While this integration can prolong antigen expression, it also raises concerns about insertion mutagenesis, disruption of normal gene function, tumorigenesis, and other unpredictable effects [116]. Therefore, future development of DNA vaccines must prioritize both safety and immunogenicity by minimizing genetic risks while improving protective efficacy.

### mRNA vaccines

mRNA vaccines involve introducing mRNA molecules that encode antigenic proteins into the human body, where they are subsequently translated into the corresponding proteins. During this process, the proteins are released from the cells to activate B cells, while intracellular processing by the proteasome generates peptides that are presented by MHC-I or -II molecules to activate CD4^+^ and CD8^+^ T cells. This mechanism promotes both humoral and cellular immune responses, offering protection against monkeypox [116].

With rapid advances in monkeypox mRNA vaccine development, several candidates have emerged (Table 3) [23, 40, 52, 136–144], which primarily differ in antigen selection and expression strategies. In terms of antigen selection, despite variations in the quantity of antigens among different vaccines, the majority use a combination of antigens from both MV and EV forms. For expression strategies, 2 main approaches exist: one involves using multiple mRNA molecules, each encoding a single antigen, which are mixed to formulate the vaccine; the other uses a single mRNA molecule encoding a fusion protein that combines multiple antigens.

The multi-mRNA strategy offers advantages such as reduced antigenic interference, precise control of protein expression levels, and flexibility in combining antigens. MV and EV proteins serve as optimal antigenic targets. M1 facilitates viral membrane integration and cytoplasmic transport, A29 interacts with the viral transmembrane protein, while A35 and B6 promote the translocation of viral particles through the ER, enhancing intercellular dissemination [57, 118, 119, 123]. Sang et al. [23] elected A29L, M1R, A35R, and B6R to develop the MPXV vaccines mRNA-A-lipid nanoparticle (LNP) and mRNA-B-LNP. Vaccination induced MPXV-specific IgG and T helper 1 (Th1) biased cellular responses in mice, conferred protection against VACV, and transferred protective immunity through their immune serum. These mice also developed long-lasting effector memory T cells and germinal center B cell responses, sustaining high-affinity antibody production. Freyn et al. [136] used the same antigens but optimized their mRNA sequences to enhance antigen expression. They modified A29 with an influenza hemagglutinin signal peptide, a de-N-glycosylation sequence, and cysteine residues. For M1, the same signal peptide was added, along with the mutation of serine/threonine to alanine, cytoplasmic domain shortening, and removal of N-glycosylation sites. For A35, the cytoplasmic and transmembrane domains were replaced with the N-terminal transmembrane domain of influenza neuraminidase. Meanwhile, the cytoplasmic tail of B6 exhibits truncation. Based on these modifications, bivalent, trivalent, and tetravalent vaccines were constructed. The tetravalent vaccine elicited higher antibody titers at lower doses than modified vaccinia Ankara (MVA) and provided enhanced Fc effector function and Th1-biased humoral immunity, leading to superior protection in VACV-challenged mice. Despite the relatively lower antibody titers for B6, its immune serum significantly delayed the spread of VACV, possibly due to inhibition of viral binding to host cells. Mucker et al. [137] further evaluated these antigens in the mRNA-LNP vaccine mRNA-1769 using a non-human primate model challenged with the MPXV Zaire 1979. Both mRNA-1769 and MVA vaccines conferred full protection, but mRNA-1769 elicited stronger humoral immune responses, increased Fc effector activity, and reduced lesion counts by 10-fold, shortened disease duration, and significantly decreased mucosal viremia. It also protected mice from intranasal and intraperitoneal MPXV infection. These findings support the ongoing Phase I/II clinical trials of mRNA-1769 (NCT05995275) [137] as a promising candidate for future outbreak control. The MV H3 protein, which binds to cell surface heparan sulfate and blocks MV adsorption [135] was included by Zuiani et al. [40] in the vaccine BNT166a (M1, H3, A35, B6) and excluded from BNT166c. Both triggered robust antibody and T cell responses, and protected mice from VACV and MPXV clade I (Zaire 79/V79-I-005) and clade IIb (hMPXV/USA/MA001/2022), and macaques from clade I (Zaire 79/V79-I-005) MPXV. A Phase I/II clinical trial (NCT05988203) is now evaluating BNT166’s safety and immunogenicity, positioning it as a leading mRNA vaccine candidate for monkeypox. Despite these successes, optimal antigen combinations remain undefined. Zhang et al. [139] developed 4 candidates (AR-MPXV5, AR-MPXV4a, AR-MPXV4b, and AR-MPXV3), all of which triggered strong antibody and Th1 responses in mice and conferred protection against VACV. AR-MPXV5 and AR-MPXV4a, which included M1R, demonstrated enhanced neutralizing antibody production, though the study did not directly compare MV- and EV-only vaccines. To address this, 4 mRNA vaccines, MPXV-E2, MPXV-M2, MPXV-M4, and MPXV-EM6, were constructed with varying combinations of MV (A29L, E8L, H3L, and M1R), EV (A35R and B6R), or different combinations of EV and MV surface proteins [139] All elicited MPXV-specific antibodies and Th1 responses. Interestingly, MPXV-M2 outperformed MPXV-E2 in neutralizing VACV, possibly due to a weaker B6R-induced IgG response in the latter. This contrasts sharply with earlier studies on subunit vaccine studies [127, 130] and warrants further investigation. Efforts to simplify production led Zeng et al. [52] to develop candidate vaccines Rmix4 (M1, A29, A35, B6) and Rmix6 (Rmix4 + H3 + E8) using a combined DNA template strategy. Both induced antibody responses, with Rmix6 showing stronger cellular immunity. In the future, this strategy will enable adjusting the proportion of different mRNA components in vaccines by changing the corresponding plasmid ratios in the DNA template mixture [139].

Although the multi-mRNA strategy reduces antigen interference, it complicates production preparation and injection. The single-mRNA fusion strategy offers simpler manufacturing and administration [142]. Hou et al. [140] designed VGPox vaccines with M1R fused to A35R. In VGPox 1 and VGPox 2, M1R was fused after the addition of a signal peptide to the extracellular domain of A35R, while VGPox 3 consisted of a mixture of full-length mRNA for M1R and A35R. Although all vaccines successfully elicited anti-A35R antibodies in mice, only VGPox 1 and VGPox 2 generated significant anti-M1R antibodies by day 7 post-immunization. In contrast, VGPox 3 did not show detectable anti-M1R antibodies until day 35. These findings suggest that the novel mRNA vaccine encoding a fusion protein of M1R and A35R triggered stronger antiviral immunity compared to a mixture of the 2 antigenic proteins. The VGPox vaccine series, based on the M1R and A35R design, thus represents a promising candidate for monkeypox prevention. Zhang et al. [139] and Zeng et al. [52] demonstrated that increasing the antigen dosNCT05995275e improves protective efficacy against both MPXV and VACV. Therefore, determining the optimal antigen quantity to achieve superior immune responses against MPXV represents a valuable and promising area of research. Fang et al. [137] designed the multivalent vaccine MPXVac-097 by sequentially linking 5 antigens (A29L, E8L, M1R, A35R, and B6R) using a 2A self-cleaving peptide. MPXVac-097 induced an imbalanced antibody response in mice, showing strong responses to E8L and A35R, while the responses to the other antigens were weaker, potentially due to interactions among the antigens. Nevertheless, MPXVac-097 demonstrated immunogenicity and potency comparable to the mRNA mixture encoding the 5 antigens (Mix-5), inducing a broad spectrum of neutralizing antibodies, MPXV-specific T cell responses, and protection against VACV challenge. Due to its simpler production process, tandem expression of antigens using 2A peptides remains an attractive strategy. To address the issue of uneven antibody responses, modifications in antigen sequence and linker design may prove beneficial [142, 143]. Ye et al. [142] also employed the 2A peptide strategy to construct 3 vaccines, LBA (B6R-A29L), LAM (A35R-M1R), and LBAAM (B6R-A35R-A29L-M1R). Unlike MPXVac-097, all 3 vaccines successfully induced antibodies against all included antigens. This improved response may be attributed to the placement of EV antigens before MV antigens, although further investigation into the underlying mechanism is necessary. Yang et al. [143] designed 2 MPXV vaccines, MPXfus and MPXmix, based on A29L, M1R, A35R, and B6R. MPXmix comprises 4 separate mRNA molecules, each encoding a different antigen protein. MPXfus expresses a fusion protein formed by connecting the 4 antigens with a flexible GGGGS linker. Both vaccines elicited comparable antibody titers and cellular immunity. However, MPXfus produced higher levels of antibodies against A35R, suggesting that the fusion of A35R with other antigens may enhance its immunogenicity.

Given the significant threat posed by orthopoxviruses such as monkeypox and cowpox to public health, the development of broadly protective vaccines is urgently needed. Hendrickson and colleagues discovered that the VACV strain contains protein-coding genes from all orthopoxvirus strains [145], and animal studies have shown that infection with one strain can confer substantial protection against others [146]. Based on these research outcomes, Su et al. [144] developed the candidate vaccine ALAB-LNP, which simultaneously expresses 4 VACV antigens: A27, L1, A33, and B5. Compared with the 4Sin-LNP vaccine, which contains a mixture of mRNAs encoding the 4 antigens separately, ALAB-LNP demonstrated higher mRNA transfection efficiency for L1 and equivalent efficiency for A33 and B5. Moreover, ALAB-LNP induced higher levels of anti-L1 IgG and similar levels of anti-A33 IgG, anti-B5 IgG, and anti-A27 IgG.

In summary, mRNA vaccines show strong efficacy and rapid development potential for monkeypox prevention, but certain limitations remain. Factors such as the integrity of the mRNA molecule, fragment length, LNP delivery system, excipient quality, and the manufacturing process can affect mRNA vaccine stability, making them prone to rapid degradation in vivo and reducing overall effectiveness. Owing to the inherent instability, mRNA vaccines often require extremely low storage temperatures, which increases transportation costs and restricts vaccine accessibility in less developed countries. The use of optimized LNP delivery systems, rigorous excipient quality control, and advanced manufacturing technologies can significantly enhance the long-term stability of mRNA vaccines [116, 147]. Furthermore, the route of vaccine administration is crucial for determining the magnitude and duration of immune response [148], and identifying the most effective delivery route remains an important area of research.

### Recombinant protein vaccines

Recombinant protein vaccines are produced by inserting protective antigen genes into prokaryotic or eukaryotic expression systems to achieve optimal expression, followed by the extraction and purification of antigen proteins or peptides. Adjuvants are subsequently added to formulate the vaccine. These vaccines contain only antigen fragments rather than the whole pathogen, rendering them especially suitable for HIV patients who cannot receive live attenuated vaccines [149, 150]. The vaccine initiates an innate immune response by activating the TLRs that recognize viral antigens or their molecular components. It also activates the MHC, which facilitates antigen presentation to T cells and amplifies the adaptive immune response [116]. Due to their high safety, strong immunogenicity, good stability, and ease of storage, recombinant protein vaccines have emerged as a promising approach for monkeypox vaccine development [116].

In the formulation of recombinant protein vaccines, the accurate selection of antigens is essential. VACV antigens serve as effective vaccine targets. For example, A33 and B5, which are antigens of the extracellular enveloped virion (EV), facilitate viral dissemination within the host via exocytosis, while L1, an antigen of the MV, is released after cell lysis to infect host cells. Fogg et al. [151] developed a vaccine targeting L1, A33, and B5 combined with the QS-21 adjuvant. This vaccine elicited MPXV-neutralizing antibodies and protected monkeys from severe disease and death. Buchman et al. [152] used the same 3 antigens to formulate a trivalent vaccine ABL, replacing the QS-21 adjuvant with cytosine-phosphate-guanine (CpG) and alum. Despite the differences in adjuvants, both the vaccine ABL and the vaccine designed by Fogg et al. [151] demonstrated comparable protective efficacy. Additionally, the study developed a quadrivalent vaccine, ABLA, by adding the A27 antigen to ABL. ABLA induced a stronger immune response than ABL, suggesting that including more antigens enhances vaccine efficacy. Notably, the IgG1 antibodies produced by ABLA can activate the complement system, which increases the vaccine’s ability to neutralize EV. This finding aligns with observations by the Crotty research group [153, 154].

Although VACV antigens are traditional vaccine targets with proven effectiveness, the emergence of the monkeypox epidemic has directed attention toward vaccines using monkeypox-specific antigens. Tang et al. [155] identified 4 structural proteins from MPXV (A29L, M1R, A35R, and B6R) and combined them with the QS-21 adjuvant. Subcutaneous administration of this vaccine on days 0, 21, and 42 led to significant increases in serum antibody levels and enhanced Th1-mediated cellular immunity in mice, protecting against sustained MPXV infection. Yang et al. [156] modified, produced, and purified the same 4 proteins to develop a recombinant protein vaccine using either alum or CpG7909 as adjuvants. This vaccine elicited strong antibody and cellular immune responses in mice, with CpG7909 demonstrating superior immunostimulatory effects compared to alum. Protein vaccines require immune adjuvants such as alum and CpG to enhance efficacy. An ideal adjuvant should both deliver target molecules and stimulate immune responses. Lin et al. [157] addressed this with a biomimetic self-adjuvanting vaccine (AM@AEvs-PB), based on A29L, M1R, and B6R. This vaccine utilizes macrophage-derived vesicles that function as adjuvants by interacting with APCs to enhance and accelerate antigen presentation while synergizing with MPXV antigens to boost immune protection.

Recombinant protein vaccines often face challenges in achieving optimal bioavailability for all immunogens, especially when co-immunized, which increases production costs and complicates industrial scaling. Wang et al. [158] addressed this issue using a structure-guided multi-antigen fusion strategy to design a “2-in-1” immunogen, named DAM, which consists of tandemly fused double A35 antigens and M1. They removed the disulfide bond in the neck region of A35 and dimerized it into a single-chain form. This approach preserved the structural integrity of the antigen while preventing heterogeneity caused by disulfide linkages during expression and purification. BALB/c mice immunized with 3 doses of the DAM-based vaccine at 3-week intervals developed similar DAM-specific IgG and VACV-neutralizing antibody titers after the second (day 40) or third (day 54) doses. The vaccine provided complete protection against lethal challenge with VACV-WR. Particularly, antibody levels induced by DAM were 28 times higher than those induced by the VACV live vaccine, and the specific antibody response was stronger than that elicited by combined immunization.

Despite the tremendous progress of recombinant protein-based monkeypox vaccines, limitations remain. One major issue is the insufficient duration of immune protection, which often necessitates multiple booster doses. The addition of adjuvants, such as aluminum hydroxide, CpG, and QS-21, can enhance immunogenicity. However, the long development cycle and high cost of production remain challenges. Future research should focus on streamlining manufacturing processes and reducing costs to improve the accessibility and effectiveness of recombinant protein vaccines for monkeypox prevention [116].

### Multi-epitope vaccines

Mutations in MPXV strains that may affect viral antigenicity and immune evasion pose challenges to vaccine design and efficacy. Conventional vaccines are often costly and time-consuming to develop and require extensive validation. In response to these challenges, multi-epitope vaccines designed using computational tools such as reverse vaccinology and immunoinformatics have gained significant attention for their potential to activate B and T cell responses. These vaccines provide long-lasting and broad immune protection against pathogens [159, 160]. Multi-epitope vaccines offer excellent safety, high specificity, and strong protective efficacy, making them advantageous in the prevention of MPXV. Compared with traditional methods, immunoinformatics can significantly reduce the time, cost, and labor required for epitope screening, while also lowering the risk of failure during vaccine development [161, 162].

Selecting MV and EV antigens that play key roles in MPXV entry into host cells is a simple and effective strategy for epitope screening [163]. MV antigen M1R and EV antigens B6R and A35R are immunogenic and essential for viral binding, fusion, and entry into target cells [164]. Suleman et al. [165] selected highly antigenic and non-allergenic CTL, helper T lymphocyte (HTL), and B cell epitopes from the protein sequences of M1R, A35R, and B6R. These epitopes were linked with a fusion adjuvant to construct a multi-epitope vaccine. The resulting vaccine exhibited a robust three-dimensional (3D) structure, proper folding, high affinity for human TLR-2, and strong immune activation. The MV protein H3L is also an attractive vaccine target, because it can induce both antibody and cellular immune responses [135]. Tan et al. [163] targeted 3 monkeypox strains from different epidemic stages and selected H3L, A35R, and B6R for epitope prediction, generating 5 vaccine candidates (MPXV-1 to MPXV-5) with different adjuvants. MPXV-2 and MPXV-5 showed broad population coverage, low free energy binding with TLRs, and stable binding to MHC molecules, suggesting that they may provide strong global protection against infection. The MV E8L protein binds to heparan sulfate on the cell surface and is located in the outer viral membrane, facilitating viral adherence. Yousaf et al. [166] performed sequence analysis of E8L and predicted B cell-derived T cell epitopes with global population coverage of 99.74%. Their multi-epitope monkeypox vaccine (MEMPV) model showed strong binding to MHC molecules, TLR3, and TLR4, and stimulated both cellular and humoral immune responses. Through sequence alignment, Shantier et al. [167] found that E8L shared 98.36–100.00% identity among MPXV variants. They selected epitopes RSANMSAPF, MSAPFDSVF, and YVLSTIHIY to construct a vaccine with strong binding to diverse MHC-I and -II alleles, suggesting broad population and protective potential. The envelope protein A28 is essential for host cell entry and viral fusion [123]. Sanami et al. [168] used sequences from E8L and A28 homologs to select CTL, HTL, and B cell epitopes. Their vaccine showed high conservation across viral strains, global population coverage of 95.57%, high antigenicity, non-allergenicity, solubility, and strong, stable binding to TLR4. Codon optimization and computational cloning confirmed high expression efficiency in the *E. coli* K12 strain. de Araújo et al. [169] selected proteins that support viral entry (L1R, A17L, H2R, A26L/A30L, and A33R), exit (A27L, A36R, A35R, and C19L), and both processes (B5R). Conservation analysis over the past 2 decades showed a 98.70% conservation rate. Epitopes that interact with BCR, MHC-I, and MHC-II were selected to create a vaccine with stable physicochemical properties, high antigenicity, and non-allergenic characteristics. This vaccine also showed strong docking with TLRs and could induce strong humoral and cellular responses.

While using well-characterized antigens is effective, a major advantage of immunoinformatic is the ability to discover novel targets through genomic or proteomic analysis [170]. For broader protection, selecting antigens from representative monkeypox strains can improve the coverage of multi-epitope vaccines [160]. Jin et al. [171] screened 10 antigenic proteins from the proteome of the MPXV-USA-2022-MA001 strain. Using appropriate linkers, they constructed a vaccine with 9 CTL, 5 HTL, and 6 B cell epitopes. The vaccine showed strong interaction with TLR2 and was efficiently expressed in the *E. coli* pET-28a (+) vector. Immune simulation indicated increases in IgM, IgG, dendritic cells, IFN-γ, and interleukin (IL). Singh et al. [172] identified good vaccine targets from the MPXV-UK_P2 strain, including C23L, C19L, F12L, I7L, G9R, L4R, A18R, and B21R. A multi-epitope vaccine was constructed using CTL, HTL, and B cell epitopes screened through unique linker coupling. Their vaccine design showed excellent stability and the potential to trigger strong immune responses. Conserved and immunodominant regions, such as envelope, membrane-bound, and extracellular proteins, were targeted to ensure broad strain coverage [160, 173]. Waqas et al. [173] used the MPXV Zaire-96-I-16 strain to select membrane-bound (E8L, A35R, A43R, C11L, D14L, E13L, G10R, B21R), extracellular (B9R, B16R), and envelope proteins (C4L). Based on the predicted B and T cell epitopes of these antigenic proteins, they designed multi-epitope vaccines. Their multi-epitope vaccines demonstrated 93.62% global coverage and 100% conservation across 6 MPXV isolates. Moreover, the epitopes were 70% to 100% homologous to experimentally validated VACV epitopes, enabling a rapid experimental stage. Aiman et al. [174] selected 3 envelope and extracellular proteins from the MPXV-USA-2022-FL001 strain and predicted 9 epitopes conserved across various MPXV strains, with full global coverage. Among the designed vaccines, MPXV-V2 is the most ideal, showing the best properties, including low energy and strong TLR4 binding. Akhtar et al. [175] studied 176 proteins from the MPXV W-Nigeria strain, selecting glycoproteins and membrane proteins. They designed a vaccine using 4 CTL epitopes, 2 HTL epitopes, 1 B cell epitope. These epitopes were highly conserved and widely distributed across global populations, effectively overcoming the limitations of antigen shift or drift. Immune simulations showed the vaccine induced IgM, IgG, IL-10, IL-12, IFN-γ, and transforming growth factor-β. Most of these vaccines target both T and B cell epitopes. However, to address the issue of T cell inhibition by MPXV [176], Pritam [177] focused on B cell epitope vaccines. Through proteomic analysis, extracellular and membrane proteins were selected, and multiple vaccine candidates were constructed. These vaccines demonstrated stability, strong antigenicity, non-allergenicity, and the ability to activate innate, cellular, and humoral immunity.

Another promising approach is pangenome-based screening, which improves the consistency of vaccine targets but also eliminates the limitations of data acquisition and selection bias [178]. Swetha et al. [178] conducted pangenome analysis on 2798 genomes, selecting outer membrane proteins (L5L, A28, and L5) based on location, solubility, and antigenicity. Among several designed vaccines, V4 showed the best immunological profile, structural quality, and strong human leukocyte antigen (HLA) binding. Alsaiari et al. [179] analyzed 19 MPXV proteomes and selected MHC-I, MHC-II, and B cell epitopes targeting the membrane protein CL5. Among the 8 candidate vaccines, V5 had the strongest interaction with HLA and TLR2/4, suggesting a robust immune response.

Despite these advancements, the design of multi-epitope vaccines faces several challenges. These include the ability to identify target antigens and epitopes, poor immunogenicity, and barriers to clinical translation. Immunogenicity may be improved by incorporating adjuvants such as cholera toxin subunit B, β-defensins, heparin-binding haemagglutinin (HBHA) proteins, and 50S ribosomal proteins L7/L12. However, most of these vaccines still lack experimental validation of their effectiveness [160].

## Novel treatments based on monkeypox antibodies

Among therapies against MPXV, antibody-based immunotherapies, including hyper-immunoglobulin (HIG), mAbs, and convalescent plasma (CP), show significant potential for treating monkeypox [180]. VIG, in particular, has demonstrated synergistic effects when used with antiviral drugs, and several mAbs have shown therapeutic promise in treating monkeypox in animal models [54, 56, 181, 182].

### Immunoglobulins

VIG, a form of HIG, is a sterile solution of high-titer anti-poxvirus IgG antibody obtained from individuals previously vaccinated against smallpox [183]. VIG acts by neutralizing both MV and EV forms of the virus. MV is primarily neutralized directly, while EV is neutralized via interactions with B5 proteins [184, 185]. Due to the high protein homology among orthopoxvirus proteins [186], VIG exhibits antiviral efficacy against VACV and variola virus (VARV), making it a promising candidate for monkeypox therapy. In response to the 2022 monkeypox outbreak and the absence of an FDA-approved treatment, the U.S. Centers for Disease Control and Prevention permitted VIG use under an emergency investigational new drug (IND) protocol. VIG was recommended for prophylactic use in individuals with a severe T cell immunodeficiency who were exposed to monkeypox [187], and its therapeutic value was subsequently validated in clinical cases [181]. VIG intravenous, a newer VIG formulation, is used prophylactically in individuals with T cell deficiencies (6000 U/kg, single dose, intravenously) and in combination with antivirals such as tecovirimat and cidofovir in patients resistant to antivirals [181, 188]. It has also been used for neonatal prophylaxis in MPXV-infected pregnant women and for treating severe neonatal monkeypox [189, 190]. Despite its promise, VIG faces challenges related to batch-to-batch variability [87]. To overcome this, Parker et al. [191] developed a recombinant VIG (rVIG) composed of 26 unique human IgG antibodies targeting different VACV proteins. rVIG consistently demonstrates superior efficacy to VIG in both in vitro and in vivo studies. In neutralization assays, rVIG exhibited approximately 2-fold lower half-maximal effective concentration (EC_50_) values and provided significant protection in mice against orthopoxvirus challenges. Both VIG and rVIG show promise for treating monkeypox, although further clinical validation is required.

### mAbs

mAbs offer an alternative to conventional VIG by targeting specific viral antigens. Their specificity, reproducibility, and defined mechanisms make them attractive therapeutic agents [184]. MV and EV differ in antigenic composition. MV-associated antigens, such as A29 and H3, are involved in viral entry into host cells, while EV-associated antigens, including B6 and A35, contribute to viral maturation and spread. Thus, mAbs can inhibit different stages of the viral life cycle, and cross-reactivity among orthopoxvirus antigens supports the use of VACV-targeted mAbs in MPXV treatment.

#### mAbs targeting antigens on MV

The A13 protein, a membrane-associated component on MV, is highly conserved among orthopoxviruses and essential for IMV production [47]. Therefore, antibodies against A13 may inhibit MPXV transmission by preventing IMV maturation. Xu et al. [53] developed the murine mAb 11F7, against A13, which reduced plaque formation by 30–40%, prevented significant weight loss, and improved survival in mice, indicating its therapeutic potential.

The A29 protein plays a role in viral adherence through interaction with acetylheparan sulfate. Li et al. [54] generated 3 mAbs against A29: 3A1, 9F8, and 2D1, with IC_50_ values of 225, 16, and 732 ng/ml, respectively. In animal models, the combination of these mAbs produced better therapeutic effects than individual administration. Differences in neutralizing activity were attributed to epitope specificity. For example, 9F8 and 3A1 bound to peptides p-1 and p-2, while 2D1 recognized p-2 and p-3. Further investigation into these mechanisms and humanization of the antibodies could enhance clinical applicability.

Nanobodies, due to their small size and variable complementary determining regions, can access hidden epitopes more effectively than conventional antibodies [192–194]. Yu et al. [195] developed nanobodies A1 and H8, with high affinity for A29. Computational affinity maturation of A1 yielded the mutant M1, which demonstrated approximately 10-fold higher affinity, likely due to the introduction of aromatic residues. Structural simulations confirmed complete binding to the heparin-binding domain of A29, highlighting nanobodies as promising therapeutics, in both the detection and treatment of MPXV.

L1, a surface protein of MV, is anchored to the poxvirus envelope and is recognized as one of the most protective antigens among poxviruses. L1 contributes to the viral entry apparatus through its interaction with the EFC and facilitates the entry of the viral core into host cells, a critical step in viral invasion and replication. mAbs targeting L1 may exert an antiviral effect by disrupting L1-EFC interactions or interfering with viral core entry into host cells. The anti-L1 mAb 7D11 has demonstrated neutralizing activity in in vitro assays, highlighting its potential for monkeypox therapy [196]. Compared to traditional antibody production using mammalian cell culture platforms, plant-based expression systems may enhance antibody efficacy by modulating Fc effector functions through N-linked glycosylation. These systems also offer advantages in terms of reduced production costs and faster manufacturing timelines [197–199]. Esqueda et al. [51] produced an anti-L1 antibody, p7D11, using glyco-engineered plants. This antibody not only effectively neutralized MPXV but also exhibited improved affinity. Remarkably, p7D11 possesses a fucosylated GnGn glycoform, which has been shown to enhance complement-dependent cytotoxicity (CDCC) and ADCC through interaction with FcγR [200, 201]. An investigation is needed to determine whether p7D11 engages additional mechanisms of viral clearance.

In summary, mAbs targeting MV surface antigens have shown promising results in both in vitro and in vivo studies, suggesting strong therapeutic potential for monkeypox. These antibodies may block viral adherence and entry into host cells, though their precise mechanisms of action remain to be fully elucidated. Additionally, nanobodies and glyco-engineered plant-derived antibodies offer novel approaches that warrant further validation in preclinical and clinical settings.

#### mAbs targeting antigens on EVs

The A33 protein is an important component of EV and can form a 3-protein complex with B5 and A34 proteins to promote EV maturation and release. Consequently, anti-A33 antibodies may inhibit viral infection by disrupting the formation of this complex, thereby hindering the maturation and release of the viral particles. The mAb A27D7 has shown high affinity for the A33 protein and demonstrated strong prophylactic and therapeutic effects against VACV and ectromelia virus (ECTV) in mice models [202]. It is noteworthy that human mAbs targeting A33 proteins tend to show higher protective efficacy than murine counterparts [182, 203]. However, developing human-derived mAbs is challenging due to the low frequency of specific B cells in vaccinated individuals, making them difficult to identify and isolate. To address this limitation, Gu et al. [182] used a multicolor antigenic tetramer technique to successfully isolate rare specific B cells and reconstruct a limited human mAb, H2. This antibody exhibited strong in vitro activity and protected mice from lethal VACV infection. Additionally, H2 IgG treatment prevented lymphocyte depletion, enhanced VACV-specific CD4 and CD8 T cell responses, and promoted antibody responses. These findings suggest that fully human antibodies targeting A33 have strong therapeutic potential against monkeypox and provide insights into their mechanism of action. Due to variability in therapeutic outcomes among mAbs targeting homologous antigens, anti-A35 antibodies may offer more promise than anti-A33 antibodies for monkeypox treatment. Meng et al. [55] developed 3 purified nanobodies targeting the A35 proteins: VHH-1, VHH-2, and VHH-3. All showed excellent specificity and affinity, with VHH-1 exhibiting the strongest binding. However, the article did not accurately predict the binding sites, leaving the reasons for differences in binding site affinity among the nanobodies unclear. Despite this, antibodies targeting A35 remain promising candidates for monkeypox therapeutics and warrant further investigation.

The B5 protein is another key antigen on EVs and a major target of neutralizing and protective immune responses. It facilitates viral entry into host cells by inducing non-lytic disruption of the EV membrane and interacts with A34 and A35 proteins to influence EV release. Therefore, antibodies targeting B5 may inhibit viral infection by blocking this membrane disruption, interfering with protein complex formation. Chen et al. [204] developed human-like mAbs, 8AH8AL and 8AH7AL, with high affinity for the VACV B5 protein. Among these, the mAb 8AH8AL effectively suppressed VACV and VARV transmission and protected mice in vivo. Further analysis showed that its binding sites on the B5 protein are highly conserved between VACV and MPXV, with potential utility in monkeypox therapy [205]. Nonetheless, antibodies developed specifically against MPXV antigens may prove more effective than those relying on cross-reactivity among orthopoxviruses. Zhao et al. [56] developed 2 mAbs, hMB621 and hMB668, targeting the B6 protein. Both antibodies neutralized VACV in vitro. In mice challenged with lethal VACV and treated with these antibodies along with complement, survival reached 100%. Mice treated with phosphate-buffered saline (PBS) lost more than 20% body weight by day 5, while hMB621 or hMB668 began to gain weight on day 4. These findings support the therapeutic potential of mAbs targeting the B6 protein for monkeypox treatment.

In conclusion, antibodies targeting EV antigens have demonstrated substantial efficacy in both in vitro and in vivo models. Since EV antigens are involved in the maturation and assembly of viral particles, these antibodies likely inhibit infection by disrupting those processes. However, the precise mechanisms of action remain unclear and require further study. Additionally, human mAbs generally show greater therapeutic potential than murine ones [202]. Given that many currently available antibodies are murine-derived, further humanization efforts represent a promising strategy to enhance their clinical utility.

#### Combination therapy with mAbs targeting antigens on MV and EV

Although treatment with a single mAb has strong potential in monkeypox therapy, combinations of antibodies targeting both MV and EV antigens generally provide superior immunoprotection. This enhanced effect is likely due to synergistic interactions between the antibodies. The major neutralizing targets include the H3 and B5 proteins. The H3 protein, located on the MV membrane, facilitates virus attachment to host cells by binding to heparin and acetylated heparan sulfate. The B5 protein, present on the EV membrane, is critical for EV membrane disruption and also participates in the maturation and release of viral particles through interactions with A33 and A34 proteins. Therefore, mAbs that target both H3 and B5 proteins may exert synergistic antiviral effects by simultaneously interfering with viral entry and the maturation process. Crickard et al. [49] demonstrated that combining anti-H3 antibody hV26 with anti-B5 antibody h101 produced significantly stronger protection against lethal VACV infections in severe combined immunodeficiency (SCID) mice than either antibody used alone. This combination also showed notable protective effects in post-exposure settings. The L1 protein, another major protective antigen on the MV surface, contributes to viral entry by forming part of the EFC and assisting the viral core’s penetration into host cells. Therefore, a combination of anti-L1 antibodies and anti-B5 antibodies may also hold great promise in monkeypox therapy. Mucker et al. [206] demonstrated that a mixture of human chimeric mAbs c7D11 (targeting L1) and c8A (targeting B5), significantly prevented MPXV infection in a nasal marmoset model. Animals that received prophylactic treatment with this antibody mixture remained disease-free during the critical period and had markedly improved survival rates. While these findings are promising, further clinical trials are needed to fully validate the potential of this combination therapy. Beyond these specific antigen combinations, other antibody pairings may also yield valuable therapeutic effects. For instance, the D8 protein, located on the MV surface, contributes to viral attachment by binding to chondroitin sulfate [50]. The combination of MV33 (targeting D8) and EV42 (targeting A33) has already shown improved efficacy in animal protection studies. This combination not only provided complete protection even when administered on day 5 post-infection but also accelerated viral clearance from target organs. In contrast, single-antibody treatments offered full protection only when administered 3 d post-infection. These results further highlight the therapeutic advantage of combination antibody treatments for MPXV.

In summary, antibody mixtures that target antigens on both MV and EV exhibit greater therapeutic efficacy than single-antibody therapies in animal models. This approach reflects the superiority of combination therapy for monkeypox treatment. However, the precise mechanisms underlying these synergistic effects remain unclear and require further investigation. Continued research into new combinations of mAbs also represents a promising direction for future development in monkeypox therapeutics.

### Nanodelivery

In addition to direct injection, mAbs can be delivered by LNPs that encapsulate mRNA-encoding specific antibodies, enabling in vivo production of antibodies. In 2022, Mucker et al. [207] successfully employed mRNAs to encode 3 specific mAbs: c8A, c6C, and c7D11, which target the B5, L1, and A33 proteins of the VACV, respectively. These mRNAs were encapsulated in LNPs and injected intramuscularly into rabbits. Although antibodies were detectable in the serum following administration, the concentrations did not reach the protective levels observed with directly administered antibodies. Consequently, Chi et al. [208] in 2024 engineered mRNAs encoding broadly neutralizing antibodies (mAbs 26, 301, 22, and 283) that target MPXV-M1, VACV-A27, VACV-A33, and VACV-B5 proteins, respectively. These mRNA constructs were administered intravenously and successfully conferred protection in a lethal VACV-WR challenge model in mice. The treatment significantly reduced pathological pulmonary lesions, validating the therapeutic potential of mRNA-encoded antibodies. Additionally, a bispecific mRNA antibody cocktail Mix2a/Mix2b, targeting both enveloped extracellular virion (EEV)-type and IMV-type forms, provided complete protection against VACV challenge in mice, and entirely prevented the development of pathological pulmonary lesions. However, the functional antibody levels in the Mix2a group were slightly lower than those achieved using single mRNA-encoded antibodies, possibly due to interference between different mRNA constructs. This observation highlights the importance of optimizing component ratios and understanding mRNA-antibody interactions to improve therapeutic efficacy. Overall, LNP-mediated delivery of mRNAs encoding human mAbs presents a promising approach for the treatment of monkeypox. While nanoparticle delivery systems are already employed in the treatment of multiple diseases, including monkeypox [209–212], other novel drug delivery platforms also hold therapeutic potential. These include bacterial derivative-mediated drug delivery, microalgae-based systems, and live-cell-based delivery systems, which have shown success in other infectious diseases and may be adaptable for monkeypox treatment [213–220].

In conclusion, antibodies against monkeypox may inhibit MPXV infection through several mechanisms. These include specific binding to viral antigens, which interferes with antigen function, and engagement with Fc receptors on immune cells to mediate antibody-dependent cellular cytotoxicity. Such mechanisms position antibodies as strong candidates for inclusion in monkeypox therapy. VIG has already been used clinically with demonstrated therapeutic benefit, although its effectiveness is limited by variability between production batches. Recombinant immunoglobulins and mAbs offer a promising alternative by overcoming these limitations. Although mAbs targeting monkeypox antigens have not yet entered clinical trials, they have shown strong therapeutic efficacy across other diseases [221]. For example, the mAb palivizumab, approved by the FDA for the treatment of respiratory syncytial virus (RSV), has been shown to significantly reduce RSV-associated hospitalizations [221, 222]. mAbs developed against MPXV or VACV have demonstrated promising outcomes in animal models, including increased survival, reduced weight loss, and decreased viral load. However, several challenges remain before their clinical application. (1) Most current in vivo protection experiments use VACV as a test virus due to laboratory constraints. The efficacy of mAbs specifically against MPXV requires further verification. (2) Clinical trials for these mAbs are still lacking. (3) The number of fully human or human-like mAbs is limited, which may lead to immunogenic reactions and reduce therapeutic efficacy in human patients. (4) The precise mechanisms of action by which mAbs interact with their viral antigens remain incompletely understood. These issues must be addressed through continued research to fully realize the potential of mAbs for MPXV therapy. Additionally, serotherapy, which has shown benefits in other viral infections such as COVID-19, has yet to be explored specifically for monkeypox and may represent another avenue worth investigating. In summary, advancing our understanding of immunotherapeutic approaches for monkeypox will aid future researchers in addressing MPXV infections and contribute to the development of novel treatment strategies. It is important to note that monkeypox treatment is not limited to immunotherapy. Photothermal and photodynamic therapy utilizing nanoparticles has already been applied in antiviral and antibacterial infections, including MPXV [223–228]. Moreover, biomimetic extracellular vesicles previously explored diseases, such as COVID-19 [229, 230], also hold potential as innovative delivery systems for monkeypox treatment.

## Challenges and future perspectives

In light of the ongoing threat posed by the monkeypox outbreak, there is an urgent need to develop improved diagnostics, vaccines, and treatments that address current limitations [231]. Antigens and antibodies are critical tools in this effort due to their proven utility in other infectious diseases, such as COVID-19 and AIDS, as well as their demonstrated efficacy in numerous animal models for prevention and treatment [232–236]. This article provides a comprehensive overview of the role of monkeypox antigens and antibodies in the development of diagnostic methods, vaccines, and treatments, offering insights into future directions for the prevention and control of monkeypox outbreaks.

Antigens of MPXV are essential to the virus’s life cycle. Antigens on the intracellular MV primarily facilitate viral entry into host cells, while those on the extracellular EV contribute to the maturation and release of viral particles [42, 237]. Notably, MV antigens such as A29L, M1R, E8L, and H3L, along with EV antigens like A35R and B6R, have advanced the development of monkeypox diagnostics and vaccines. However, several challenges remain. Current antigen-based diagnostic approaches primarily rely on the A29 protein. Although these methods demonstrate high sensitivity and specificity, they remain prone to false-negative results. Exploring alternative antigens may improve diagnostic accuracy and reliability [90]. Moreover, there is still a limited understanding of the mechanisms of MPXV antigens, and the 3D structures of many of these proteins have not yet been resolved. Further investigation is essential for advancing vaccine and therapeutic development.

In vaccine development, several critical issues merit deeper exploration. First, the source of the antigen is a key consideration. Most current DNA vaccines are designed using antigens from VACV [127, 131–133], and some recombinant protein and RNA vaccines also rely on VACV-based designs [144, 151, 152]. For example, Hooper et al. [132] developed a 4-antigen DNA vaccine based on A27L, L1R, A33R, and B5R, while Hirao et al. [133] designed a multivalent DNA vaccine containing 8 antigens (A27, F9, H3, L1, A33, B5, A56, and A4). In contrast, many mRNA vaccines are developed using MPXV antigens. Although both design strategies have been effective in animal models, no direct comparison has yet determined whether MPXV-derived or VACV-derived antigens provide stronger protection. Therefore, selecting the appropriate antigen source is essential for future vaccine development. Second, novel antigens should be evaluated alongside those already under investigation. Current nucleic acid and recombinant protein vaccines predominantly target 6 well-known MV and EV antigens. Although these are involved in key viral processes such as entry, attachment, and transmission, the antigenic potential of other MPXV proteins remains underexplored. Bioinformatics approaches offer a safe and time-efficient means to identify promising new antigens. Once validated experimentally, these antigens can be incorporated into vaccine designs and evaluated for their immune response profiles and clinical relevance compared to existing antigens. Third, optimizing the number of antigens included in a vaccine formulation is a pressing question. Research suggests that increasing the number of antigens can improve immune efficacy, but this conclusion is primarily based on studies of a limited set of antigens. As more candidates are identified, it becomes important to determine the minimum number of antigens required to achieve optimal protection. Excessive inclusion may lead to immune tolerance or diminish immune tolerance or diminish the immune response to clinically important targets [148]. Vaccines like the single-immunogen DAM offer valuable insights into minimal antigen design and may inform broader platform development beyond recombinant proteins [158]. The ratio of antigens used in vaccine formulations also warrants consideration. By adjusting antigen ratios based on their individual roles and immunogenic properties, vaccines may achieve improved protective outcomes [52]. Another unresolved issue is the durability of vaccine-induced antibody responses. Long-term monitoring is needed to evaluate whether these vaccines provide lasting protection against potential variations in MPXV. While traditional smallpox vaccines offer long-lasting immunity [238, 239], the duration of protection from current monkeypox vaccines remains unclear. Most DNA, mRNA, and recombinant protein vaccines show strong humoral and cellular immune responses in animal models, but only BNT166 has progressed to phase I/II clinical trials. The rest remains in preclinical stages. Accelerating their transition into clinical practice is critical to meet the current public health needs. In parallel, ongoing training for healthcare professionals, public health education, and awareness initiatives are necessary to ensure effective deployment and uptake. Mathematical and statistical modeling tools have been developed to assess vaccine distribution strategies and contact tracing. For instance, Bankuru et al. [240] proposed a game-theoretic model to evaluate vaccination programs.

Antibodies targeting MPXV antigens are essential not only for diagnostics but also for the development of effective immunotherapies. Despite the advantages of speed and high sensitivity, there are several challenges associated with the use of antibodies in antigen detection. One major obstacle is the difficulty in obtaining high-affinity antibodies during the screening and capture process. Future research should focus on generating more specific and high-affinity antibodies will be a promising direction to improve detection sensitivity and enhance test performance. Strategies such as improving antigen delivery systems, incorporating adjuvants, and using computer-aided design of antibodies may be useful tools to address this problem [98, 195]. Moreover, detection technologies based on antibodies often involve time-consuming and complex screening procedures. [94]. Streamlining these processes is critical to accelerating the development of novel diagnostic tools. Therapeutically, antibodies have demonstrated substantial potential across multiple animal models. Numerous monoclonal and polyclonal antibodies have shown strong efficacy both in vitro and in vivo. Furthermore, cowpox immunoglobulin has shown encouraging results in clinical settings. Despite the promise of antibody-based immunotherapy, several practical challenges hinder its widespread application. First, most currently available antibodies are murine mAbs, which face significant clinical limitations due to the risk of immune rejection. Therefore, further development of humanized antibodies is necessary to fully validate their clinical potential. Second, the specificity of antibodies for a single epitope limits their effectiveness. To address this issue, the advancement of antibody combination therapies, as well as the development of bispecific or tri-specific antibodies, represents a promising research direction [49, 50]. Third, many mAbs have yet to be fully validated for efficacy against MPXV. This is often due to constraints in laboratory conditions, highlighting the need for further experimental validation. Fourth, although antibodies developed using various platforms have shown binding activity, their mechanisms of action remain unclear [51]. It is still unknown whether antibodies produced on different platforms retain neutralizing activity or other in vivo antiviral mechanisms such as CDCC and ADCC. Finally, logistical and financial barriers to the production and distribution of antibody-based therapies remain a significant concern, particularly in low-income countries and regions. These challenges include: 1) high manufacturing costs due to complex processes, reliance on cell culture platforms, and stringent storage requirements, all of which reduce accessibility in low-income countries; 2) a limited and geographically concentrated global supply of mAb drugs, which fails to meet clinical demands in low-resource regions; 3) weak transportation infrastructure in low-income regions, further restricting access [241–243]. To overcome these challenges, several strategies should be pursued: (1) Public and private sectors must innovate to reduce the cost of mAb production. (2) Alternative production methods should be explored. For example, plant-based expression systems can yield high biomass yield without the need for expensive fermentation infrastructure. Additionally, mRNA-encoded neutralizing antibodies present a promising option for scalable and cost-effective production [244]. (3) Expanding manufacturing capabilities to underserved regions such as Latin America and Africa should be a priority. (4) Governments could implement pooled procurement strategies to negotiate lower prices and improve access to advanced therapies. (5) Sustained investment in local infrastructure and capacity building will be critical to overcoming logistical obstacles and improving epidemic preparedness [242, 243, 245, 246].

While scientific insights into monkeypox antigens and antibodies are promising for outbreak containment, translating these findings into targeted public health interventions remains slow. High-risk groups, including LGBTQI communities and individuals living in poor housing conditions, face multiple barriers that require comprehensive responses [247, 248]. These include the establishment of effective surveillance systems, the establishment of real-time case reporting, rapid diagnostics, contact tracing, education for healthcare providers, vaccine optimization, and equitable access to treatments [248]. Our structural characterization of monkeypox antigens and their corresponding antibodies provides a strong foundation for the development of 1) next-generation diagnostics with enhanced sensitivity (detection limit < 10^4^ PFU/ml) and rapid turnaround times (less than 30 min); 2) safer vaccine platforms with improved immunogenicity; and 3) mAbs with superior efficacy for both pre-exposure prophylaxis and post-exposure treatments. Although novel antigen-based monkeypox detection technologies offer advantages in speed, convenience, and sensitivity, their real-world application requires further validation. Essential steps include performance testing in the field, operator training, and widespread dissemination in communities. Integration with existing surveillance platforms, such as China’s Notifiable Disease Reporting System and the US National Notifiable Diseases Surveillance System (NNDSS), can streamline the introduction of these tools into public health programs through digital data uploading. In terms of vaccination strategies, there is an urgent need to develop and deploy safer, more cost-effective vaccines that can be mass-produced. Although many vaccines based on monkeypox antigens have shown promising protective results in animal studies, multiple steps remain before they can be widely used in high-risk populations. These include additional animal testing, clinical trials to confirm safety and efficacy, manufacturing scale-up, post-marketing surveillance, and efficient distribution. Resources such as ClinicalTrials.gov (United States), the WHO International Clinical Trials Registry Platform (ICTRP), and the European Union Clinical Trials. Register can be used to monitor the design, status, and results of vaccine trials. mAbs targeting monkeypox antigens offer renewed hope for treatment, as animal experiments have confirmed their protective effects. However, further studies are necessary to evaluate their safety, optimize delivery methods, and improve manufacturing and distribution. Only by addressing these challenges can mAbs become a viable tool for protecting vulnerable communities during monkeypox outbreaks [249].

While efforts to contain the monkeypox outbreak continue, it is critical to prioritize ethical considerations during the accelerated development of vaccines and therapeutics. These include ensuring safety, obtaining informed consent, and promoting equitable access. Although rapid research and development are necessary in emergencies, fundamental safety principles must be upheld. This includes rigorous identification of potential risks and continuous safety monitoring throughout all clinical phases. Informed consent must be genuinely voluntary and free from coercion or undue inducement. Furthermore, intervention strategies such as modes of administration and follow-up procedures, as well as communication methods including choice of messengers, channels, and messaging content, should be tailored to align with local cultural norms and values. At the societal level, preparedness is essential. Surveys have indicated a widespread lack of knowledge among the general public and healthcare providers, regarding monkeypox symptoms, diagnosis, and treatments [250, 251]. Moreover, the current concentration of cases among men who have sex with men, including gay and bisexual men, has led to harmful stigmatization. It is imperative to provide enhanced support services to these high-risk communities while safeguarding their dignity and well-being. Public health interventions must be designed to avoid perpetuating stigma or discrimination against LGBTQI populations [251]. Politically, national authorities must prioritize high-risk settings prone to rapid infectious disease transmission. This includes establishing and implementing effective containment measures that both respect public rights and ensure national readiness. Upon the emergence of disease outbreaks, national early warning systems should be activated immediately. These systems must notify relevant stakeholders and prompt the coordinated execution of national protocols for early detection, case and contact management, rapid risk assessment, as well as consistent reporting updates [248]. At the international level, disparities in epidemic response between countries remain a significant concern. Addressing these disparities requires long-term strategies, including substantial investment in the healthcare systems of endemic regions such as those in Africa. These efforts are essential for achieving global health equity and improving overall epidemic preparedness [252–256].

In conclusion, monkeypox antigens and antibodies demonstrate substantial potential for preventing and managing monkeypox infections. Diagnostic technologies based on antigen and antibody detection offer high sensitivity and rapid results. They also require minimal laboratory infrastructure and training, making them especially useful in field settings. Unlike serological diagnostics, these methods can detect infection without needing a defined window period for antibody development, thereby enabling earlier diagnosis. Therapeutic strategies that utilize antibodies are promising for the development of targeted monkeypox treatments and may offer particular advantages for immunocompromised individuals or those with drug-resistant infections. Likewise, vaccines formulated with monkeypox antigens hold significant potential in the absence of a licensed monkeypox-specific vaccine. These vaccines can precisely target immunogenic viral proteins, induce both humoral and cellular immune responses, and may be particularly effective for protecting high-risk and immunocompromised populations. However, key knowledge gaps remain. The detailed molecular architecture and functional mechanisms of monkeypox antigens are not yet fully understood. Similarly, the precise viral clearance mechanisms mediated by antibodies require further elucidation. Continued research is essential to resolve these uncertainties and to develop innovative strategies for the prevention and control of monkeypox.

## Data Availability

Not applicable.

## Abbreviations

3D: Three-dimensional
AaLS: Aquifex aeolicus lumazine synthase
ADCC: Antibody-dependent cytotoxicity
AIDS: Acquired Immunodeficiency Syndrome
APCs: Antigen-presenting cells
AuNP: Gold nanoparticle
CCR: C-C chemokine receptor
CDCC: Complement-dependent cytotoxicity
COVID-19: Coronavirus disease 2019
CP: Convalescent plasma
CpG: Cytosine-phosphate-guanine
CTL: Cytotoxic T lymphocyte
CXCR: C-X-C chemokine receptor
DRC: Democratic Republic of the Congo
ECTV: Ectromelia virus
EFC: Entry/fusion complex
ER: Endoplasmic reticulum
EV: Enveloped virion
FDA: Food and Drug Administration
GAGs: Glycosaminoglycans
HBHA: Heparin-binding haemagglutinin
HIG: Hyper-immunoglobulin
HLA: Human leukocyte antigen
HTL: Helper T lymphocyte
ICA: Immunochromatographic assays
ID: Intradermal injection
IEVs: Intracellular enveloped viruses
IFN: Interferon
IL: Interleukin
IM: Intramuscular injection
IMV: Intracellular mature virion
IND: Investigational new drug
IV: Immature virion
LFIA: Lateral flow immunoassay
LNP: Lipid nanoparticle
LOD: Limits of detection
mAb: Monoclonal antibody
MEMPV: Multi-epitope monkeypox vaccine
MHC: Major histocompatibility complex
MoO3: Molybdenum trioxide
MPXV: Monkeypox virus
MV: Mature virion
NK: Natural killer
PCR: Polymerase chain reaction
QDs: Quantum dots
rVIG: Recombinant VIG
RSV: Respiratory syncytial virus
SCID: Severe combined immunodeficiency
SCS: Sandwich coupling switch
SERS: Surface-enhanced Raman scattering
SIV: Simian immunodeficiency virus
TCR: T cell receptor
TGN: Trans-Golgi network
Th1: T helper 1
TLR: Toll-like receptor
TRFIA: Time-resolved fluorescence immunoassay
VACV: Vaccinia virus
VARV: Variola virus
VIG: Vaccinia immunoglobulin
WHO: World Health Organization
